# Efficient identification of somatic mutations in acute myeloid leukaemia using whole exome sequencing of fingernail derived DNA as germline control

**DOI:** 10.1038/s41598-018-31503-5

**Published:** 2018-09-13

**Authors:** Purvi M. Kakadia, Neil Van de Water, Peter J. Browett, Stefan K. Bohlander

**Affiliations:** 1 Leukaemia & Blood Cancer Research Unit, Department of Molecular Medicine and Pathology, The University of Auckland, Auckland, New Zealand; 2 LabPlus, Department of Diagnostic Genetics, Auckland City Hospital, Auckland District Health Board, Auckland, New Zealand

## Abstract

Recent advances in next-generation sequencing have made it possible to perform genome wide identification of somatic mutation in cancers. Most studies focus on identifying somatic mutations in the protein coding portion of the genome using whole exome sequencing (WES). Every human genome has around 4 million single nucleotide polymorphisms (SNPs). A sizeable fraction of these germline SNPs is very rare and will not be found in the databases. Thus, in order to unambiguously identify somatic mutation, it is absolutely necessary to know the germline SNPs of the patient. While a blood sample can serve as source of germline DNA from patients with solid tumours, obtaining germline DNA from patients with haematological malignancies is very difficult. Tumor cells often infiltrate the skin, and their DNA can be found in saliva and buccal swab samples. The DNA in the tips of nails stems from keratinocytes that have undergone keratinization several months ago. DNA was successfully extracted from nail clippings of 5 probands for WES. We were able to identify somatic mutations in one tumor exome by using the nail exome as germline reference. Our results demonstrate that nail DNA is a reliable source of germline DNA in the setting of hematological malignancies.

## Introduction

Acute myeloid leukemia (AML) is a devastating disease with very poor survival, especially in elderly patients^1^. AML is caused by genetic changes like chromosomal rearrangements or point mutations that are somatically acquired in haematopoietic stem cells (HSC). In contrast to chronic myeloid leukaemia (CML), which is almost exclusively caused by the t(9;22)(q34;q11) translocation^2^ resulting in the BCR/ABL fusion gene^3^, AML is driven by a great number of different somatic chromosomal abnormalities and point mutations^4^. More than 130 different fusion genes have been described so far in AML^5^ with 79 different fusions involving the *KMT2A* gene alone^6^. However, this cytogenetic diversity in AML represents just the tip of the iceberg, i.e. only those genetic changes that can be detected by conventional cytogenetics, which has a resolution of about 5 to 10 Mbp

With the advent of next-generation sequencing (NGS) it has become possible to analyse AML genomes at single base pair resolution to identify somatic point mutations. AML whole genome sequencing (WGS), whole exome sequencing (WES) and targeted sequencing studies have discovered that about 6–26 somatic single nucleotide variants (SNVs) are present in protein coding genes^7,8^ of which 5–10 SNVs alter the amino acid sequence. Recent studies analyzing 68 genes in 664 AML samples found that, on average, 4–5 mutations are observed in every AML patient^9^. Our ability to identify oncogenic genetic rearrangements and somatic driver mutations in AML is essential for precise diagnosis, prognostication, minimal residual disease (MRD) monitoring and might inform treatment decisions including targeted treatment^10^.

WES is more economical than WGS and offers genome wide coverage compared to gene panel sequencing. Therefore, WES is often the preferred approach for exploratory studies of cancer genomes. Typically, WES identifies about 18,000 to 20,000 nucleotide positions that are different from the reference genome, so called single nucleotide variants (SNVs), in the 54 Mbp covering the coding region of the human genome. Close to 95% of these SNVs are known, harmless single nucleotide polymorphisms (SNPs), described in the various SNP databases. After removing known SNPs, about 99% of the remaining 600–1000 SNVs are also non-pathogenic germline SNPs which are specific to an ethnic group or the family of the proband and which have not been deposited in any of the SNP databases yet. Thus, fewer than 0.1% of the SNVs are actually tumor specific somatic variants. In order to identify these tumor specific somatic variants confidently, it is essential to know the germline SNPs of the proband. These can be identified by performing WES on DNA from a non-tumor tissue sample from the same proband (matched germline control).

While it is easy to obtain a germline DNA sample, e.g. peripheral blood (PB) DNA, from patients with solid tumors, it is very challenging to obtain germline DNA from patients with hematological malignancies^11^. Sources for germline DNA in such patients are often not available (like stored pre-malignant PB or bone marrow (BM) samples or remission PB or BM samples), difficult and inconvenient to obtain (e.g. skin biopsies) and/or have a high potential to be contaminated with malignant blood cells (e.g. PB, buccal swab or sputum samples). A less commonly used source for germline DNA are finger or toenails.

Here we report the identification of somatic mutations in AML using fingernail DNA as germline reference. We extracted DNA from fingernail clippings of an AML patient and successfully used this DNA to unambiguously identify somatic mutations in the DNA extracted from a leukemic BM sample of the same patient at the time of diagnosis.

## Materials and Methods

### Patient samples

Bone marrow and nail DNA for this study was from a patient (P1) with a myelodysplastic syndrome (MDS) which progressed into acute myeloid leukemia who was treated at Auckland City Hospital. Since biallelic mutations in *CEBPA* were found, which are not usually seen in MDS, additional diagnostic, genetic testing was requested by the treating physicians. Four additional patients (P2 to P5) provided paired nail and peripheral blood or bone marrow samples for genetic testing of other conditions. Informed consent for genomic testing was obtained from all patients according to the guidelines of the Auckland District Health Board. The consent form and patient information sheets used were obtained from and approved by the Human Genetics Society of Australasia (www.hgsa.org).

### DNA extraction

The DNA from the bone marrow (BM) and blood samples was isolated on the MagNA Pure instrument (Roche, Pleasanton, CA, USA). The protocol for the nail DNA extraction was adapted from a user developed protocol for the isolation of genomic DNA from nails and hair with the QIAamp® DNA Mini Kit (Qiagen, Hilden, Germany). For the isolation of DNA from fingernails, 3–5 fingernail clippings (15–30 mg) were cut into small pieces of about 1 mm^2^ followed by a quick wash with 70% ethanol and two successive washes with distilled water. The nail pieces were then incubated overnight at 55 °C in 300 µL of digestion buffer (100 mM NaCl, 10 mM EDTA, 10 mM Tris-HCl pH 8.0, 40 mM DTT (dithiothreitol), 2% SDS and 2.5 µg/µL proteinase K; proteinase K and DTT were added to the buffer just before use). The next day, the DNA was extracted using the QIAamp DNA mini kit (Qiagen) and spin protocol for blood samples. We used 300 µL of buffer AL (200 mM Tris-HCl pH 7.5, 25 mM EDTA pH 8.0, 0.5% SDS, 250 mM NaCl) and ethanol instead of the recommended 200 µL to maintain the 1:1 ratio with the sample buffer. The DNA was finally eluted in 200 µL of buffer AE (10 mM Tris-HCl, 0.5 mM EDTA pH 9.0). The DNA quantification and quality assessment was performed using Qubit (Invitrogen), agarose gel electrophoresis and/or on a Tapestation 2200 (Agilent Technologies). The quality of the nail DNA was also assessed by PCR amplification of a genomic region.

### Whole exome sequencing (WES)

Nail and BM gDNA (250 ng each) were sheared using the EpiShear™ Multi-Sample Sonicator (Active Motif). The condition used to obtain fragments in the size range of 100–400 bp was 65% amplitude, 2 to 3 rounds of 20 minutes with alternating 30 sec on and 30 sec off (total 20–30 minutes on time). Before each cycle, the temperature of the sonicator was ensured to be at 4 °C. The quantity and the fragment size of the sheared DNA was assessed on a Tapestation 2200 (Agilent Technologies) with the high sensitivity D1000 tape. 100 ng of sheared DNA was used for the preparation of the whole exome libraries (WEL). WELs were prepared using the SureSelect XT2 (SSXT2) reagent kit and the SureSelect Clinical Research Exome V2 exome enrichment kit (Design ID #S06588914) following the manufacturer’s instructions (Agilent Technologies). The WEL were sequenced on a NextSeq. 500 (NCS v2.0, Illumina Inc.) to obtain around 40 to 44 million paired end reads (2 × 150 bp or 2 × 75 (P3 and P4-nail), 4.3 to 14.4 Gbp) per exome.

### WES Data analysis

The quality of the sequences was assessed using Fastqc (https://www.bioinformatics.babraham.ac.uk/projects/fastqc/). The sequences were aligned to the human reference genome (hg19) with BWA (bwa 0.7.12)^12^. The resulting sam files were converted to bam and then the bam files were sorted using the samtools (Samtools-1.3.1)^13^. Mpileup files were generated (Samtools 1.3.1) with the following parameters: maximum depth (-d) 500, minimum base quality (-Q) 15 and minimum mapping quality (-q) 10. In order to call the somatic variants in the tumor (BM) sample, the somatic function of Varscan v2.3.9^14^ was used for the germline mpileup (nail) and the tumor mpileup (BM) files to generate VCF (variant call format) files^15^. The somatic variants in the vcf files were annotated with information from various SNP databases (dbSNP138 etc) using ANNOVAR^16^ followed by the annotation for the variants’ effect with SnpEffect^17^. Somatic or loss of heterozygosity variants predicted to have a ‘High’ (e.g. non-sense) or ‘Moderate’ (missense) impact and with a somatic p value of ≤0.001 were selected using SnpSift^18^. Variants present in dbSNP (142) or the 1000genome_Oct2014 database were excluded.

### WES Data quality assessment

To assess the quality of the data and efficiency of the target enrichment procedure, hybrid selection, per target coverage, insert size, alignment summary and GC bias metrics were generated using picard-tools-2.4.1 (http://picard.sourceforge.net).

All experimental procedures and methods were performed and carried out according to the guidelines and regulations of the Auckland District Health Board and the University of Auckland.

## Results

### DNA Quantity and quality

**Figure 1:**
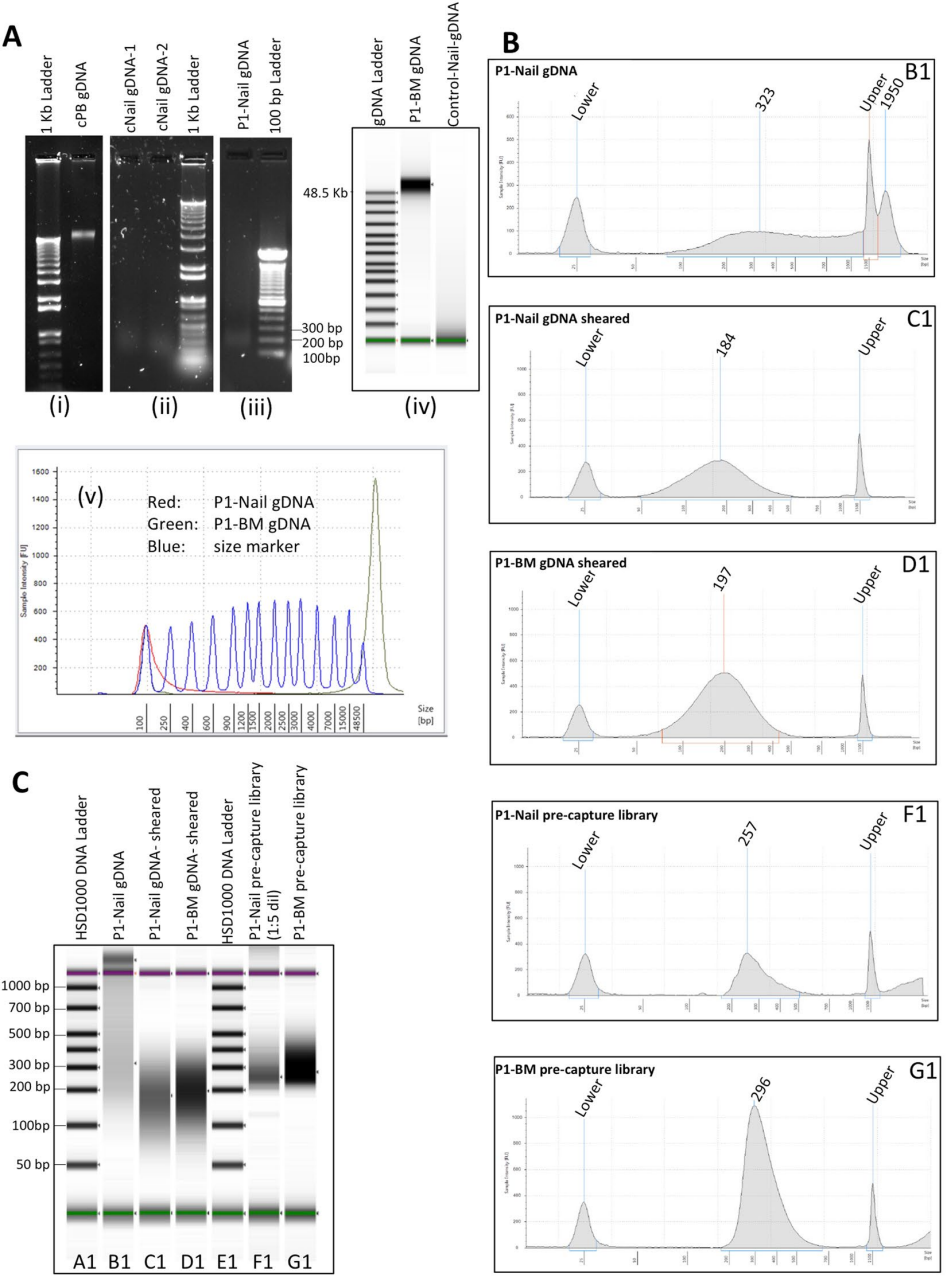
DNA quality: **(A)** (i-iii) SYBR green I stained agarose gels showing DNA extracted from control peripheral blood (i: cPB gDNA), from control nail clippings (ii: cNail) and from patient 1 nail clippings (iii: P1-Nail). (iv,v) Tapestation genomic DNA tape gel picture (iv) and peak traces (v), comparing the bone marrow DNA from patient 1 and the nail DNA from P1. (**B**) Tapestation traces showing the non-sheared nail gDNA (trace B1), sheared nail gDNA (C1), sheared BM gDNA (trace D1) and amplified indexed gDNA libraries from the nail (trace F1) and BM DNA (trace G1) from patient 1. (**C**) Tapestation gel of the traces in (**B**). Note: the nail gDNA library (trace F1) was run at a 1:5 dilution.

The quantity and quality of the extracted DNA from the nail and BM samples from patient 1 (P1) were assayed using Nanodrop, Qubit, agarose gel electrophoresis and a TapeStation (Agilent) (Table 1, Fig. 1A(i-iv)). We obtained 516 ng DNA from 20 mg of nail clippings from P1 based on Qubit measurements. An average of around 18 ng of DNA per 1 mg of nail clippings (range 7 to 40 ng DNA/mg nail) could be extracted in a series of 8 samples (Supplementary Table 1). As expected, the bone marrow sample had a much higher DNA yield (about 10 µg from 1.6 × 10^6^ cells). In addition, striking differences in quality of the DNA extracted from nail and BM were observed. The nail DNA was highly fragmented with the fragments ranging in size from around 100 bp to about 2 kbp (Fig. 1A(ii-v),B), while the DNA from the BM sample was running in the compression zone on the gDNA tape indicating good quality, high molecular weight DNA with fragments greater than 15 kbp (Fig. 1A(iv)). To confirm that the quality of the nail DNA from P1 was sufficient for the downstream library preparation steps we successfully amplified a 197 bp genomic DNA fragment from the *PBGD* gene from this DNA (data not shown).

**Table 1:** DNA quantification.

Sample ID	NanoDrop (ng/µL)	NanoDrop (A260/A280)	Qubit (ng/µL)	TapeStation (ng/µL)	Total DNA in 200 µL***
P1-BM	91.2	1.89	51.4	42.0*	10.28 µg
P1-Nail	13.2	2.13	2.58	1.09**	516 ng

*Measured on gDNA tape, **measured on high sensitivity D1000 tape. ***Based on Qubit measurements.

### Whole exome library preparation and sequencing

Even though the nail DNA was very fragmented, the size distribution of the fragments was quite large and many fragments were bigger than the recommended fragment size (around 300 bp) for NGS library preparation (Fig. 1B, trace B1; Fig. 1C, lane B1). Therefore, we sheared both the bone marrow and the nail DNA by sonication. Interestingly, despite being already quite fragmented, the nail DNA required almost the same sonication intensity and duration as the high molecular weight BM DNA to obtain fragments in the recommended 100 bp to 400 bp size range (Fig. 1B, trace C1 and D1; Fig. 1C, lanes C1 and D1).

The sheared nail and the BM DNA samples were then used for the Sureselect XT2 gDNA library preparation and exome capturing procedure. The DNA yields after the standard library preparation procedure of the nail and BM libraries for P1 were 216 ng and 165 ng, respectively (Fig. 1B, trace F1 and G1; Fig. 1C, lanes F1 and G1). Thus, it can be inferred that the quality of the nail DNA was as good as that of the BM DNA for the NGS library preparation. We saw similar results when we prepared the peripheral blood and nail WES libraries for the other four patients (P2-P5). After exome capturing, the libraries were sequenced on a NextSeq 500, allocating 40 million clusters of paired-end reads (2 × 150 bases) to each exome library.

### Sequencing output and quality metrics

Sequence quality was evaluated with the fastqc tool, which showed that more than 74.5% of the bases from the nail and more than 78.8% of the bases from the bone marrow exomes from P1 had PHRED scores above 30. After aligning the reads to the human reference genome (hg19) with BWA, quality metrics were generated using Picard tools. Alignment summary metrics after removing PCR duplicates showed that more than 107 million and 106 million passed filter (PF) reads (all paired) were obtained from the nail and BM exome libraries of P1, respectively (Table 2). The proportion of the PF reads aligned to the hg19 reference genome from the P1 nail WES was slightly lower than that from the BM WES: 97.2% in nail vs 98.3% in BM. However, almost all the aligned PF reads aligned in pairs both in the nail (99.4%) and in the BM (99.5%) exomes. These results indicated that the quality of the sequence obtained from the nail and the BM DNA was comparable (Table 2). Similar results were obtained from 4 additional patients for which we performed paired WES on DNA extracted from peripheral blood (PB) or BM and nail (Table 2). The percentage of PCR duplicates in the nail exomes was, on average, higher (29% to 56%) than in the PB of BM sequences (19% to 46%) indicating that the nail libraries had a lower complexity than the PB or BM libraries. In P3 there were slightly more PCR duplicates in the BM exome sequences than in the nail exome sequences.

**Table 2:** Whole Exome Sequencing metrics from PICARD.

Hybrid Selection Metrics/ Sample Info	P1-BM	P1-Nail	P2-PB	P2-Nail	P3-BM	P3-Nail	P4-PB	P4-Nail	P5-PB	P5-Nail
**Ref genome**	hg19	hg19	hg19	hg19	hg19	hg19	hg19	hg19	hg19	hg19
GENOME_SIZE	3,101,804,741	3,101,804,741	3,101,804,741	3,101,804,741	3,101,804,741	3,101,804,741	3,101,804,741	3,101,804,741	3,101,804,741	3,101,804,741
BAIT_TERRITORY	54,098,923	54,098,923	54,098,923	54,098,923	54,098,923	54,098,923	54,098,923	54,098,923	54,098,923	54,098,923
TARGET_TERRITORY	35,495,642	35,495,642	35,495,642	35,495,642	35,495,642	35,495,642	35,495,642	35,495,642	35,495,642	35,495,642
PF_UNIQUE_READS	106,789,318	107,344,759	94,279,613	50,395,498	69,779,721	62,557,872	77,039,161	67,576,894	72,182,769	65,553,926
PCT_PF_UQ_READS	100.0%	100.0%	100.0%	100.0%	100.0%	100.0%	100.0%	100.0%	100.0%	100.0%
**PCT_PF_UQ_READS_ALIGNED**	**98**.**3%**	**97**.**2%**	**97**.**7%**	**95**.**3%**	**97**.**8%**	**95**.**2%**	**98**.**1%**	**97**.**2%**	**98**.**2%**	**99**.**3%**
**MEAN_TARGET_COVERAGE (fold)**	**79**.**68**	**74**.**24**	**93**.**42**	**46**.**92**	**32**.**26**	**31**.**12**	**74**.**84**	**32**.**84**	**78**.**21**	**62**.**74**
MEDIAN_TARGET_COVERAGE	77	72	94	42	28	28	72	30	74	15
**FOLD_ENRICHMENT**	**16**.**94**	**17**.**04**	**22**.**94**	**27**.**04**	**19**.**75**	**22**.**19**	**22**.**51**	**21**.**07**	**25**.**04**	**24**.**12**
ZERO_CVG_TARGETS_PCT	1.6%	0.8%	1.9%	2.2%	6.2%	2.9%	2.2%	3.4%	1.8%	21.0%
**PCT_TARGET_BASES_20×**	**87**.**0%**	**91**.**6%**	**86**.**8%**	**69**.**4%**	**58**.**2%**	**60**.**4%**	**83**.**2%**	**61**.**0%**	**84**.**6%**	**48**.**6%**
**Alignment Summary Metrics**										
Total # of paired PF Reads	106,789,318	107,344,759	94,279,613	50,395,498	69,779,721	62,557,872	77,039,161	67,576,894	72,182,769	65,553,926
# of PF Reads aligned (to the Ref genome)	104,974,657	104,325,201	92,111,167	48,019,798	68,231,575	59,539,607	75,591,666	65,685,799	70,875,401	65,097,422
**% of PF reads aligned (to the Ref genome)**	**98**.**3%**	**97**.**2%**	**97**.**7%**	**95**.**3%**	**97**.**8%**	**95**.**2%**	**98**.**1%**	**97**.**2%**	**98**.**2%**	**99**.**3%**
PF_ALIGNED_BASES	14,427,132,005	13,107,271,771	12,506,043,273	5,375,447,023	5,092,009,746	4,367,257,183	10,208,827,634	4,851,700,895	9,610,230,332	8,182,262,500
PF_HQ_ALIGNED_READS	100,823,219	99,200,185	88,407,559	44,853,721	64,978,905	55,761,327	72,476,022	62,059,678	68,327,276	62,267,394
PF_HQ_ALIGNED_BASES	13,937,375,831	12,567,096,599	12,092,852,199	5,127,395,157	4,863,529,709	4,118,092,143	9,863,025,210	4,604,831,212	9,319,611,775	7,885,746,592
PF_HQ_ALIGNED_Q20_BASES	12,525,100,793	11,286,362,421	10,946,285,477	4,783,558,180	4,682,608,199	3,973,604,910	8,910,130,015	4,441,395,882	8,462,020,636	7,380,096,882
**MEAN_READ_LENGTH**	**142**	**131**	**141**	**118**	**75**	**74**	**141**	**75**	**141**	**131**
**READS_ALIGNED_IN_PAIRS**	**104**,**420**,**518**	**103**,**689**,**169**	**91**,**553**,**864**	**47**,**878**,**460**	**67**,**693**,**047**	**59**,**049**,**484**	**75**,**100**,**061**	**65**,**166**,**522**	**70**,**349**,**086**	**64**,**968**,**572**
**PCT_READS_ALIGNED_IN_PAIRS**	**99**.**5%**	**99**.**4%**	**99**.**4%**	**99**.**7%**	**99**.**2%**	**99**.**2%**	**99**.**3%**	**99**.**2%**	**99**.**3%**	**99**.**8%**
**MEAN_INSERT_SIZE**	**175**	**141**	**172**	**122**	**184**	**128**	**170**	**136**	**170**	**139**
**PCT Duplicates**	19.3	28.8	41.7	49.1	45.7	44.5	38.3	43.8	39.3	56.0

Note that the metrics shown in Table 2 were calculated after PCR duplicates had been removed.

**Figure 2:**
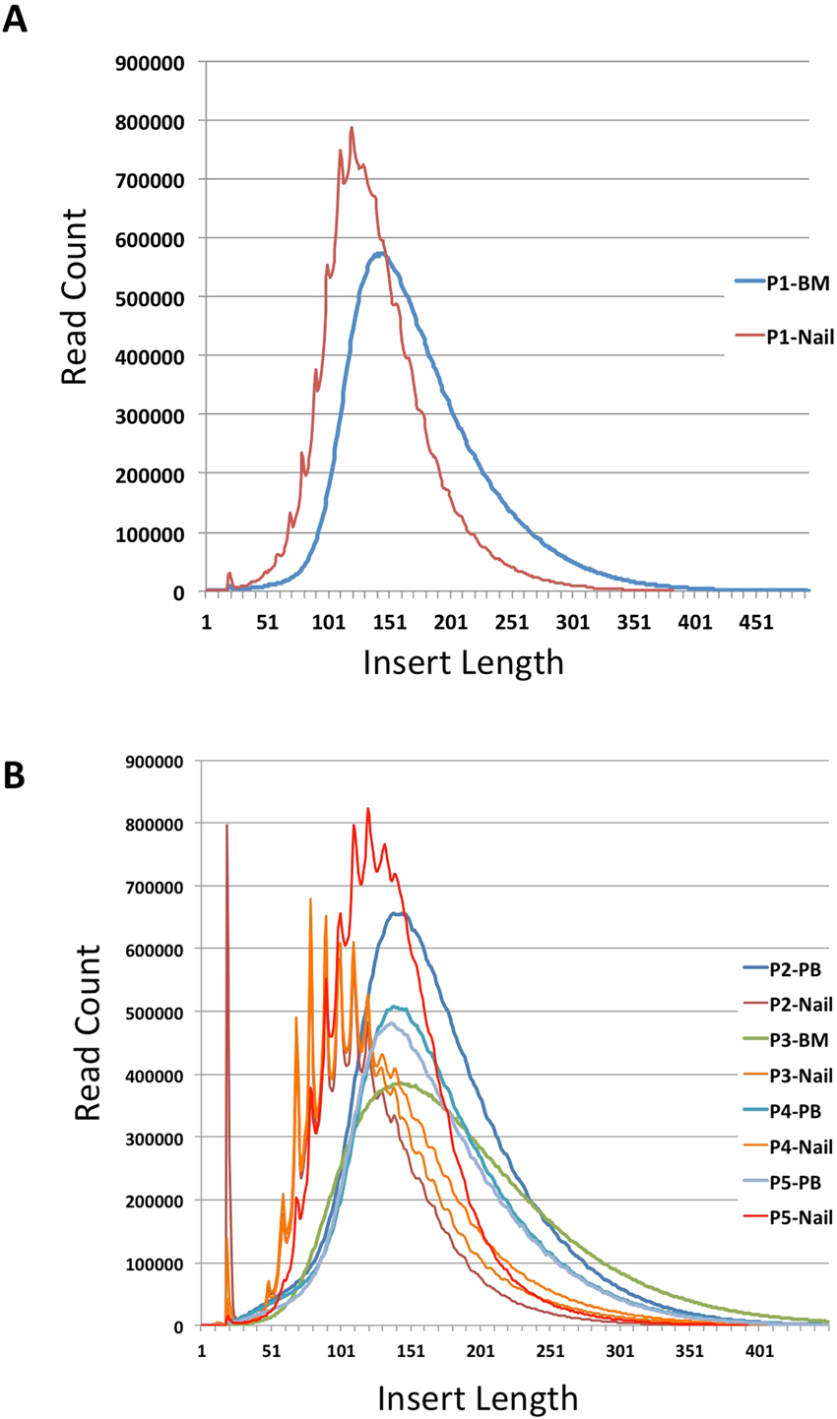
Insert size histograms of BM/PB and nail libraries: **(A)** P1 BM and nail library; **(B)** PB or BM and nail libraries from patients P2, P3, P4 and P5. Note the saw-tooth pattern in the histogram of the nail samples and the prominent spike at an insert size of 19 and 20 bp in the P2 nail sample.

The average insert length (±standard deviation) of the P1 nail whole exome library (WEL) was with 140.9 ± 43.92 bp slightly shorter compared to that of the BM WEL (175 ± 56.11 bp). In the other four paired sample, we saw a similar pattern with the PB/BM libraries having average insert lengths between 170 and 184 bp, while the insert size lengths in the nail libraries ranged from 122 to 139 bp (Table 2). Interestingly, we observed a peculiar saw tooth pattern in the insert size histogram of the nail WEL with a periodicity of about 10 bp (Fig. 2A). This saw tooth pattern was even more prominent in the four other nail WELs which we have analyzed (Fig. 2B). There was a prominent spike at 19 and 20 bp in the distribution of insert sizes from the P2 nail library.

To assess the quality of the exome capturing experiment, hybrid selection and per target coverage metrics were calculated. The capture baits regions provided by the manufacturer of the capturing probes (SSXT2: Design ID # S06588914, Agilent) were used as bait interval (BI: 54.1 Mbp). As target interval (TI) we used the regions in common between the capture baits and the conserved coding DNA sequences from hg19 (CCDs exons) with 10 base pair padding into the neighbouring introns (TI: 35.5 Mbp). For the patient P1, the mean bait coverage was 72.1 and 78.9 fold, and the mean target coverage was 74.2 and 79.7 fold for nail and BM, respectively. The target enrichment was 17 fold for nail and BM. 91.6% and 87.0% of the target region were covered at a depth greater than 20 fold in the nail and BM library, respectively (Table 2).

### Identification of somatic variants

**Figure 3:**
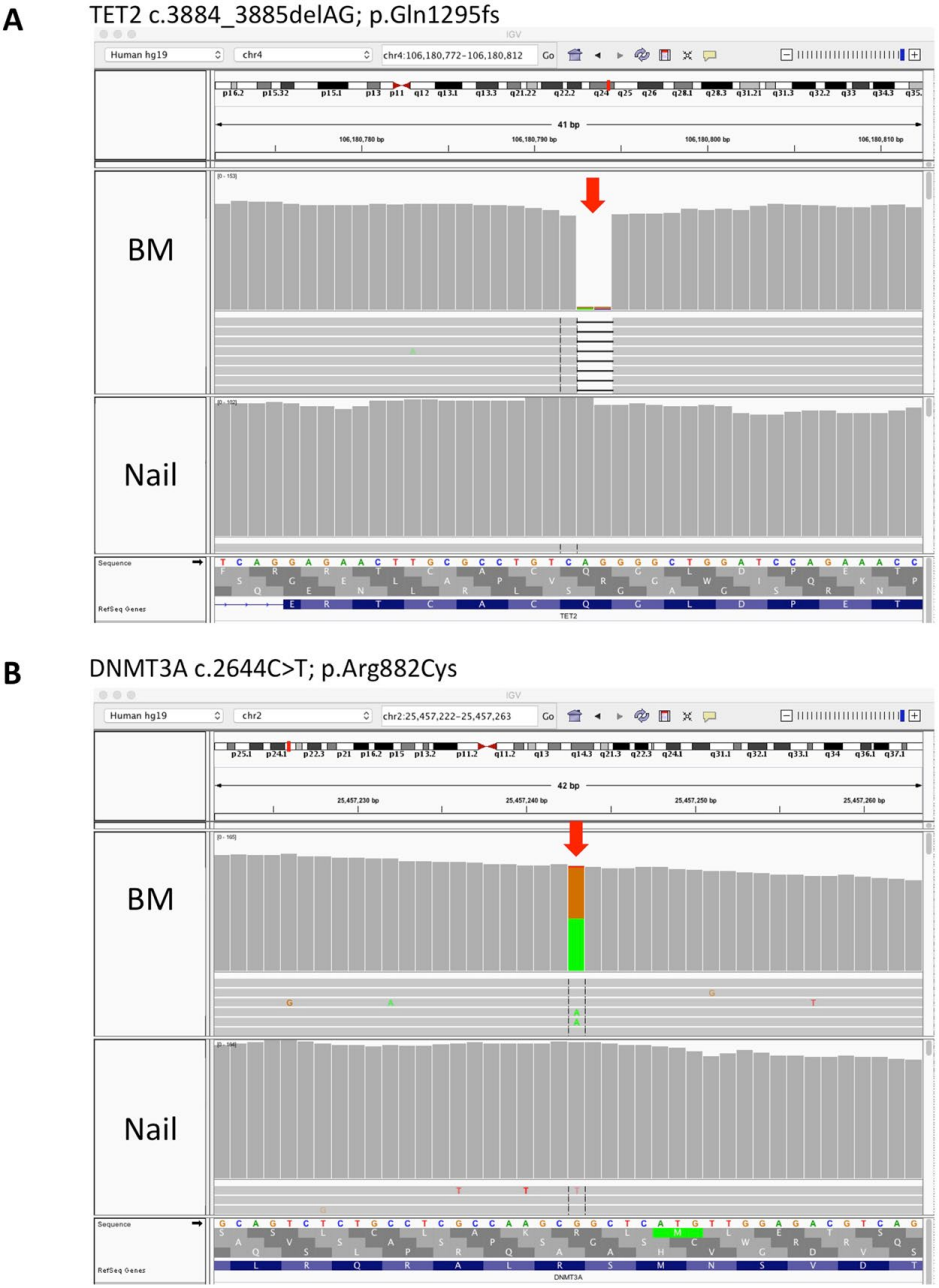
Integrative Genomics Viewer screenshots: **(A)** 2 basepair *TET2* deletion: c.3884_3885delAG; p.Gln1295fs (arrow). **(B)** somatic *DNMT3A* single nucleotide variant: DNMT3A c.2644 C > T; p.Arg882Cys (arrow).

The main aim of our experiments was to test whether nail-derived DNA can be used as germline reference to identify somatic variants in acute myeloid leukemia. We used the Varscan somatic algorithm with the nail exome as germline and the BM exome as tumor to call putative somatic variants in the BM exome sequence of P1. As parameters for the Varscan somatic algorithm the normal purity was set at 100% and tumor purity to 90%. The resulting variants were annotated with the information from various single nucleotide polymorphism databases using Annovar and the effects of the variants was predicted using SnpEff. SnpSift was used to filter the VCF files using as parameters a somatic P-value of ≤0.001, a variant allele frequency of >20%, a minimum read depth of 25, more than 5 reads supporting the variant in the tumor, and fewer than two variant reads detected in the germline. In P1, 11 somatic variants with high (frame shift or nonsense) or moderate (missense) predicted impacts on the encoded proteins were detected. These included 10 point mutations and one 2 bp deletion (Table 3). Examples of somatic variants in *DNMT3A* (p.Arg882Cys) and *TET2* are shown as screenshots from the Integrative Genomics Viewer (IGV) (Fig. 3). Interestingly, the *DNMT3A* p.Arg882Cys variant was clearly a somatic variant, but it was annotated as a single nucleotide polymorphism in dbSNP142 (rs377577594). This variant is also listed as the most common somatic variant in *DNMT3A* in the COSMIC database. Missense variants in three other genes (*CACNA1S*, *TTN* and *STAB1*) had dbSNP142 entries and very low (0.0002) allele frequencies in the 1000 genomes database. However, they appear to be true somatic variants in P1. Six other genes had somatic missense variants, including *SPI1*, which is a critical, lineage determining transcription factor in haematopoiesis^19^, and *UPF3A*, a regulator of non-sense mediated mRNA decay (Table 3).

**Table 3:** Somatic Variants.

	Chr	Position	Somatic P-Value	GT TU	VAF GL	VAF TU	RR GL	RR TU	AR GL	AR TU	Gene	cDNA change	Protein change	DB_SNP142 ID	Allele frequency 1000 genomes
1	1	201021733	1.64×10^−12^	0/1	0%	48.0%	64	38	0	35	*CACNA1S*	c.3905 G > A	p.Arg1302Gln	rs200042281	2.00 × 10^−04^
2	11	47376923	1.06 × 10^−08^	0/1	1.32%	37.1%	75	39	1	23	*SPI1*	c.671 T > C	p.Met224Thr	none	0
3	13	115057253	4.79 × 10^−04^	0/1	0%	34.3%	26	23	0	12	*UPF3A*	c.832 G > T	p.Glu278*	none	0
4	18	24496554	3.29 × 10^−10^	0/1	0%	38.6%	70	43	0	27	*CHST9*	c.1001 T > A	p.Ile334Asn	none	0
5	2	25457243	3.76 × 10^−13^	0/1	1.35%	52.4%	73	30	1	33	*DNMT3A*	c.2644 C > T	p.Arg882Cys	rs377577594	0
6	2	159992708	7.15 × 10^−07^	0/1	0%	43.2%	38	25	0	19	*TANC1*	c.263 C > T	p.Pro88Leu	none	0
7	2	179425063	5.27 × 10^−14^	0/1	0%	40.7%	84	67	0	46	*TTN*	c.85796 G > A	p.Arg28599His	rs558543425	2.00 × 10^−04^
8	3	52538536	4.97 × 10^−09^	0/1	0%	47.2%	46	28	0	25	*STAB1*	c.1210 G > A	p.Val404Ile	rs200927449	2.00 × 10^−04^
9	6	26045721	3.31 × 10^−05^	0/1	1.85%	34.3%	53	23	1	12	*HIST1H3C*	c.83 A > T	p.Lys28Met	none	0
10	8	104897909	1.68 × 10^−14^	0/1	0%	52.5%	65	38	0	42	*RIMS2*	c.416 G > A	p.Arg139Gln	none	0
11	4	106180792	2.24 × 10^−28^	1/1	0.0%	90.1%	55	7	0	64	*TET2*	c.3884_3885delAG	p.Gln1295fs	none	0

Legend: GT: genotype, GL: germline, TU: tumor, VAF: variant allele frequency, RR: reads supporting reference, AR: reads supporting variant.

Note: all the GTs in GL were 0/0.

A homozygous deletion in *TET2* (p.Gln1295fs) resulting in a frameshift was also exclusively observed in the BM sample (Fig. 3A, Table 3). As can be seen in the histograms of the IGV screenshots in Fig. 3A and in Table 3, while almost all reads had the 2 bp deletion in the BM (leukemia) exome, there were no reads supporting the 2 bp *TET2* deletion and only one read showing the *DNMT3A* point mutation in the nail DNA (Fig. 3B, and read counts in Table 3), confirming the usefulness of nail sample as a source of germline DNA for the identification of somatic variants in leukemia samples. That the quality of the sequence from the nail DNA is comparable to quality of sequences obtained from PB or BM extracted DNA and allowed for the reliable calling of germline variants, was also apparent from the absence of database annotated common SNPs that were called as somatic variants. Only three putative somatic single nucleotide variants had an annotation in dbSNP142. However, all three of these SNPs had a very low allele frequencies in the 1000 genome database and might represent annotation mistakes, mapping mistakes or variants from clonal haematopoiesis of indetermined potential (CHIP) in dbSNP142 (like rs377577594 in *DNMT3A*), and could in fact be true somatic variants in P1.

The two somatic variants in the *CEBPA* gene in P1 that were found in our routine diagnostic Sanger sequencing of the *CEBPA* gene were not found in our WES analysis. This was due to the near zero read coverage of the high GC content regions of *CEBPA* where these mutations were located.

## Discussion

With recent advances in next-generation sequencing technologies, sequencing costs have dropped to the point where it has become possible to perform large scale analysis of complete tumour exomes and genomes^20^. Most cancers are caused by the accumulation of several somatic driver mutations. In many cancers, including haematological malignancies, there are a great number of genes that can be mutated and almost every cancer has a different combination of driver mutations. For example, it is well established that in most acute myeloid leukaemia samples, about 4 to 8 driver mutations in any combination of more than 200 genes are found^21^. These somatic mutations have an impact on prognosis and might also be important in tailoring treatment to the specific cancer^4^. It is thus very important to unambiguously identify somatic mutations. However, due to the great number of genes that can harbour potential driver mutations, it is often difficult to distinguish somatic mutations from rare polymorphisms. One can only be certain that a given variant is a true somatic mutation if it is not present in the germline DNA sample from the same patient. While peripheral blood is a readily accessible source of germline DNA in the case of solid tumours, it is difficult to find a germline DNA source that is reliably and consistently free of tumour DNA contamination in the case of haematological malignancies, especially for leukaemias. For example, in one of the first examples of whole genome sequencing of a leukaemia where DNA extracted from a skin biopsy was used as germline reference, it was noted that there was infiltration of leukemic cells in the skin of the patient as tumour specific variants could also be detected in the presumptive germline sample^22^.

In the present work, we show that nail clippings are a good source of germline DNA that enables the unambiguous identification of somatic variants in patients with leukaemia. As can be seen (Fig. 3B, Table 3), there was not a single read among 55 reads in the nail DNA that showed the two basepair deletion in the *TET2* gene which was present in 64 of 71 reads (VAF of 90%) in the leukaemia sample. *TET2* loss of function mutations are well known recurring driver mutation in AML^23^. The number of somatic mutations (11 in this study) detected in the AML sample falls within the range of the number of driver mutations reported in the literature for this disease^9^. However, our WES analysis did not discover the somatic *CEBPA* mutations that were present in P1 due to the near zero read coverage of the high GC content exon of *CEBPA*. It is therefore important to keep in mind that the sensitivity of mutation discovery in WES is extremely poor in certain genomic regions. Another factor that might limit the power to discover somatic variants in the tumor sample can be contamination of the tumor DNA with germline DNA. In patient P1, the contribution of germline DNA in the tumor sample was less than 10% since the 2 bp deletion of *TET2* has a VAF of 90%, which suggest a tumor purity of about 90%.

DNA extracted from nails has been used in the past, especially in forensics, to identify individuals^24^ but also for SNP genotyping studies^25^. In these settings only a limited number of unique loci, mostly microsatellite or short tandem repeats, was PCR-amplified and typed. A few studies have also shown that nail DNA is adequate next-generation sequencing of smaller panels^26^ and also for large scale genotyping (>262,000 loci) on array platforms^27^. We show for the first time, to our knowledge, that sufficient DNA, both in terms of quality and quantity, can be extracted from nail clippings to perform WES, thus enabling the identification of germline variants in all human genes. Although the DNA obtained from the nail samples was rather fragmented with a size range of 100 bp to 2 kbp, the DNA was of sufficient length for successful NGS library preparation. In fact, the nail DNA had still to be sonicated to obtain the preferred size for NGS library preparation.

The amount of DNA that can be extracted from ten nail clippings (i.e. the nails from two hands), even when normalized to the input nail weight, varied from sample to sample but we obtained sufficient DNA (200 to 500 ng) in every case to perform WES. The DNA yield could probably be increased if the nail clippings are mechanically ground into finer pieces before proteinase K digestion as we noticed that nail fragments were still present after overnight proteinase K digestion.

There has been one report that the DNA from fingernails can be derived from donor cells in bone marrow transplant recipients, indicating that haematopoietic cells can contribute to the DNA that is found in fingernails^28^. However, as can be seen in Fig. 3B and Table 3, there was no obvious contribution of the malignant haematopoietic clone to the fingernail DNA in our patient at the depth of coverage achieved in our experiment (about 60 to 100x). The somatic variants in *SPI1*, *DNMT3A* and *HIST1H3C* had one read supporting the somatic variant in the nail sample (at read depths of 54 to 76). Whether these single variant supporting reads are a sign of DNA from the malignant haematopoetic clone is unclear. Thus, we cannot exclude contributions of the malignant clones at low levels of less than about 2%. There are two, not mutually exclusive, explanations for why there was no obvious contribution of the malignant clone to the fingernail DNA in our sample. The first is that the DNA obtained from fingernail clippings (i.e. the tip of the fingernails) is derived from keratinocytes that underwent keratinization several months before the malignant clone developed. Especially in the case of acute myeloid leukaemia, the growth of the malignant clone just before diagnosis is quite rapid, so that one has to assume that the DNA at the tips of the fingernails was deposited long before the leukemic clone expanded. The other explanation is that the situation in the bone marrow transplant recipient described in the report by Imanishi and colleagues^28^ is quite different from the situation in a patient with AML. Bone marrow transplant patients receive an intense conditioning regiment before their transplant. The conditioning regimen is toxic not only to bone marrow cells and haematopoietic stem cells (HSC) but to all cells in the body. It is therefore quite likely that some skin stem cells are also killed by the conditioning regimen. In this situation, some of the infused donor HSCs might be attracted to the partially empty skin stem cell niches and transdifferentiate into skin stem cells that generate the keratinocytes at the roots of the nails. This would explain why donor derived polymorphisms can later be found in DNA from nail clippings of BM transplant patients. Since the skin stem cells are not compromised during leukaemia development, it seems very unlikely that leukemic stem cells will take up residence in the full skin stem cell niche in the nail bed and differentiate into keratinocytes for nail growth.

We noticed that the size distribution of the insert size of the nail exome library was not smooth like the distribution of the insert size from the bone marrow exome. There was a saw tooth pattern in the size distribution histogram of the nail exome with a 9 to 10 bp periodicity (Fig. 2). We were unable to find a good explanation for this 9 to 10 bp periodicity. It is unclear whether this periodicity is a result of the fragmentation process of nuclear DNA during keratinization, whether it is the result of preferential breakage of DNA during the sonication or an artefact of the library preparation process. However, this periodicity corresponds to the number of bases in one turn of the DNA double helix. It is known that during the process of keratinzation, the DNA wrapped around nucleosomes is degraded amongst others by DNASE1L2^29^. If this or other DNAses preferentially cleave the DNA backbone that is not protected by the nucleosome, it could result in a 9 to 10 bp periodicity in the size distribution of the resulting DNA fragments.

The availability of a reliable source of germline DNA in the form of nail-derived DNA will facilitate paired germline-tumor WES at the diagnosis of leukaemia. Such an approach will make it easier to discover rare leukemia-predisposing germline variants and also rarer somatic mutations. In contrast to gene panel sequencing, a WES approach would be more ‘future-proof’ as the data sets can be reanalyzed at later timepoints as more information becomes available about germline susceptibility genes and about important somatic mutations. A WES approach would obviate the need to redesign gene panels frequently. Even though a greater sequencing depth can be achieved with gene panels, the somatic mutations in the major clones at presentation will be detectable at the sequencing depth of WES.

We conclude that DNA extracted from nail clippings is an excellent source of germline reference DNA for the unambiguous identification of somatic mutations in haematological malignancies when performing whole exome sequencing. We have also used nail DNA to unambiguously identify somatic mutations by targeted gene panel sequencing. In our hands, nail-derived DNA did not show any contamination with the malignant clone, even when about 90% of DNA extracted from bone marrow was of malignant origin. A further advantage is that nail clippings, both from fingers and toes, are easy to collect and can be transported and stored at room temperature for long times without further degradation of the DNA.

## Electronic supplementary material

Supplementary Information Overview

DataSet 1

DataSet 3
