# Supplementary material for: Efficient identification of somatic mutations in acute myeloid leukaemia using whole exome sequencing of fingernail derived DNA as germline control
Source: Sci Rep. 2018 Sep 13;8:13751. doi: 10.1038/s41598-018-31503-5 (PMC6137150; doi:10.1038/s41598-018-31503-5)
Supplement: Supplementary file 1 — Supplementary Information Overview [file 41598_2018_31503_MOESM1_ESM.docx]

**Supplementary Information**

**Manuscript SREP-18-06301A**

**Efficient identification of somatic mutations in acute myeloid leukaemia using whole exome sequencing of fingernail derived DNA as germline control**

Purvi M. Kakadia^1^, Neil Van de Water^2^, Peter J. Browett^1^, Stefan K. Bohlander^1^

^1^Leukaemia & Blood Cancer Research Unit, Department of Molecular Medicine and Pathology, The University of Auckland, Auckland, New Zealand, ^2^ LabPlus, Department of Diagnostic Genetics, Auckland City Hospital, Auckland District Health Board, Auckland, New Zealand

**Overview of Supplementary files**

**DataSet 1**

**Supplementary Table 1 (DNA yields)**

NailMS_SREP-18-06301_SupplementaryTable1

**DataSet 2**

**Original uncropped gel image files:**

Fig1Ai_original.tiff see below

Fig1Ai-ii-iii_original.tiff see below

**DataSet 3**

**Original PICARD insert size metrics output to generate Fig. 2**

Nail_PaperMetricsWorkbookJan2018RevisionMay2018.xlsx

**DataSet 4**

**Patient Consent and Patient information sheet (blank copies)**

General Genomic Consent.pdf DataSet 4-1

Patient_Information_sheet.pdf DataSet 4-2

**Fig1Ai_original.tiff**

**
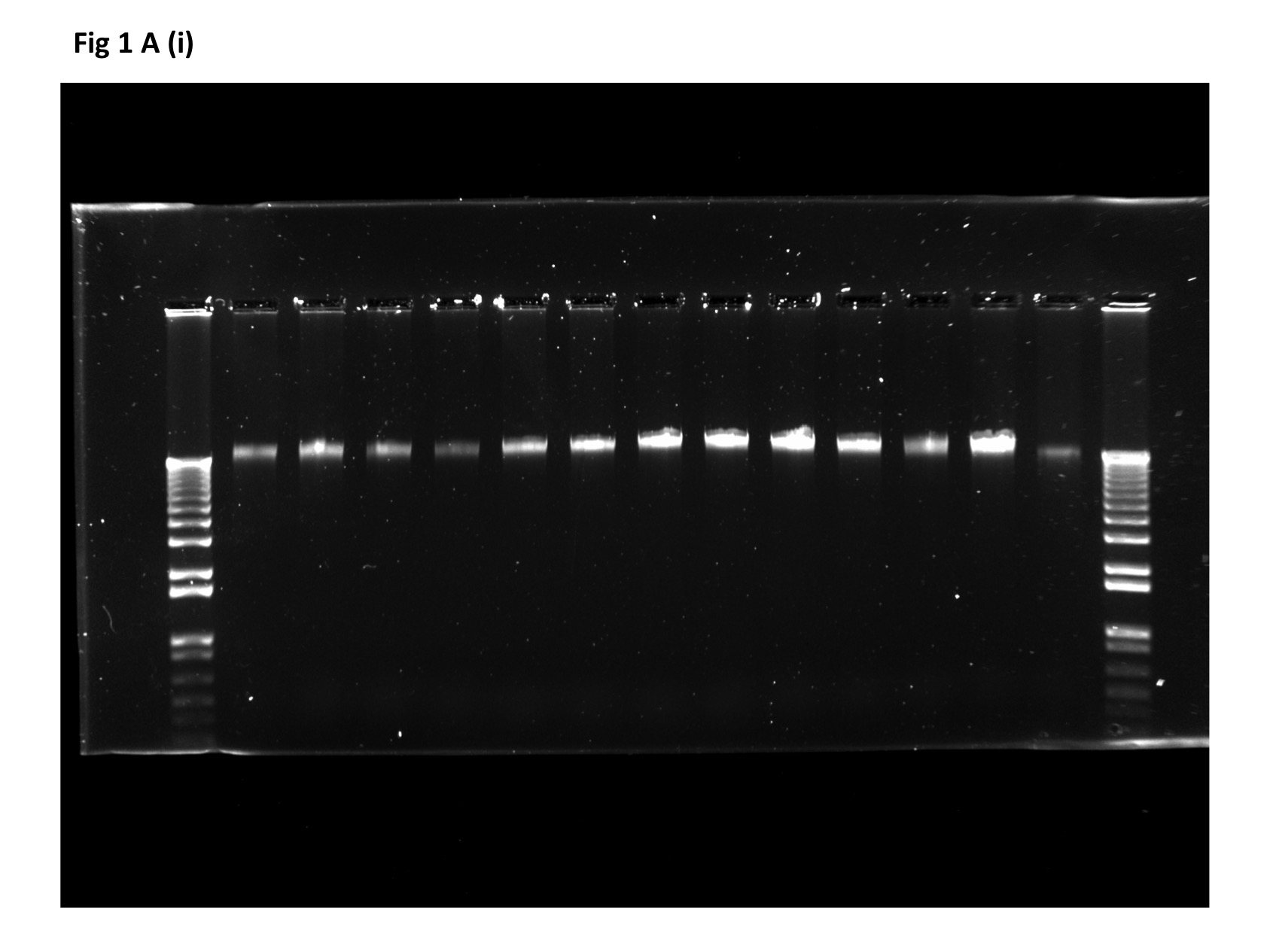
**

**Fig1Ai-ii-iii_original.tiff**

**
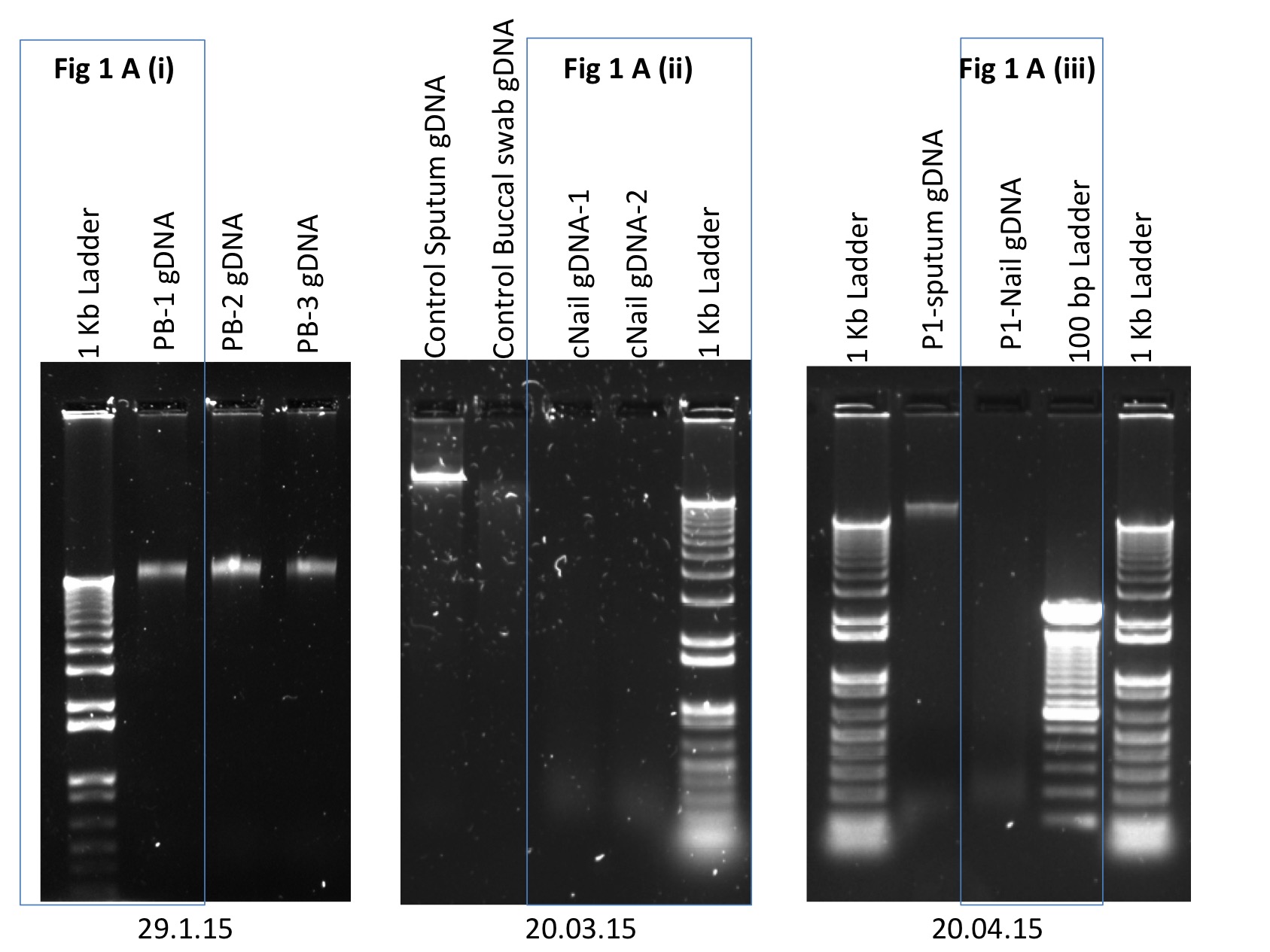
**
